# Low-Dose radiation risk in medicine: a look at risk models, challenges, and future prospects

**DOI:** 10.1016/j.zemedi.2025.07.002

**Published:** 2025-07-25

**Authors:** Forough Jafarian-Dehkordi, Christoph Hoeschen

**Affiliations:** Chair of Medical System Technology, Institute for Medical Technology, Faculty of Electrical Engineering and Information Technology, Otto von Guericke University, Germany

**Keywords:** ionizing radiation, radiation risk, risk models, radiation protection, uncertainty

## Abstract

The application of ionizing radiation in medical diagnostics and treatments has been transformative in advancing healthcare for the benefit of patients. However, with these advancements comes the need to understand and mitigate the risks of ionizing radiation. While the role of radiation in medicine is undeniable, its potential to induce malignancies and genetic alterations requires careful application and understanding. This review provides an overview about the radiobiology of radiation risk, and then go through the evolution, challenges, and inherent uncertainties surrounding radiation risk models. In the end, it looks at the impact of the technological and methodological progress that has influenced the radiation protection and shapes our understanding of radiation risk. The search for references was conducted in Google Scholar and PubMed using the keywords ’low radiation,’ ’radiation risk,’ ’risk models,’ ’radiation protection,’ and ’uncertainty.’

## Introduction

Radiation protection is essential in medical diagnostics and treatments. With the increasing use of radiological methods in contemporary medicine, understanding and reducing the dangers of ionizing radiation has become paramount. Each exposure to radiation, even if minimal, potentially carries an inherent risk. This risk, primarily the potential for malignancies or genetic alterations, emphasizes the need for careful use of radiation in medical environments. The primary goal is to maximize the benefits of radiation while minimizing its risks to patients and medical staff.

Risk models are important in the field of radiation safety, offering a structured approach to predict health implications from radiation exposure. For instance, the Linear No-Threshold (LNT) model, as the commonly used model, translates radiation doses into quantifiable health risks. These models are helpful in establishing radiation safety standards, directing medical procedures, and shape health regulations. They provide a connection between radiation exposure metrics and decisions based on evidence, ensuring healthcare professionals prioritize patient safety.

This review explores radiation risk models and their scientific foundations, examines historical progression of radiation protection practices, and discusses the uncertainties and challenges associated with risk models’ application in radiological protection and risk management. It is important to clarify that this review excludes radiotherapy from its scope, as the focus is on low-dose radiation exposure in diagnostic imaging. By concentrating on diagnostic applications, the review aims to shed light on the evolving strategies and advancements that enhance our understanding and management of radiation risks in this particular context.

## Underlying Radiobiology of Radiation Risk

The interaction of ionizing X-ray and gamma photons with living cells initiates a cascade of damage and defense processes. Primary ionizations can directly break chemical bonds in critical biomolecules like DNA, but, at low doses, indirect effects via water radiolysis dominate. In this process, X-rays and gamma photons excite and ionize water molecules within milliseconds, generating reactive oxygen species (ROS) such as hydroxyl radicals (·OH) [1]. These ROS diffuse to attack DNA bases, sugars, and other cellular components, contributing to single-strand breaks (SSBs) and base lesions. Immediately, cellular antioxidant defenses start to neutralize radicals within seconds to minutes, mitigating the initial oxidative burst. Enzymatic antioxidants (superoxide dismutase, catalase) and glutathione can neutralize radicals within seconds to minutes, mitigating the initial oxidative burst [1]. At very low doses of low-LET radiation, the contribution of ROS-mediated effects may outweigh that of direct DNA ionizations, although this balance can vary depending on radiation quality and biological context [1].

Even 1–100 mGy can induce dozens of SSBs and a few double-strand breaks (DSBs) per cell [1]. DNA damage sensing and repair mechanisms have been reported to activate within minutes of radiation exposure, though the timing may vary depending on cell type and conditions. Cells usually detect DSBs via sensor proteins like Ataxia-Telangiectasia Mutated (ATM), triggering the p53 signaling pathway and cell-cycle checkpoints. High-fidelity repair pathways then reverse the damage: base excision repair remedies base lesions and SSBs, while DSBs are rejoined by non-homologous end-joining or homologous recombination. At low doses, these repair processes are generally efficient and may help reduce the likelihood of mutation propagation in many cases [2]. Post-exposure, transcription of certain repair genes and synthesis of proteins like TCTP (in a p53- and p21-dependent manner) have been observed to increase within hours after low-dose exposures (≤100 mGy), which may enhance cellular resilience in some contexts [[3], [4], [5]]. However, evidence for a sustained increase in overall repair capacity remains mixed and appears to depend on factors such as dose level, cell type, and timing. This dynamic response suggests that cells may induce additional repair activity as a potential adaptive mechanism in the low-dose regime.

Cellular defensive responses extend beyond repair to manage damage on immediate and delayed timescales. Instantly, metabolic defenses neutralize ROS, and heavily damaged cells may undergo apoptosis or senescence to self-eliminate [6,7]. Over hours to days, gene expression programs bolster defenses, activating the Nrf2 antioxidant pathway and increasing stress-response protein production [2]. These adaptive responses peak later, conferring heightened protection against subsequent injuries. Some changes, such as epigenetic modifications or long-lived repair proteins, have been observed to persist for days or weeks following exposure [2]. While such changes may contribute to genome stability, their functional significance remains complex and is still under active investigation. In this way, low-dose irradiation may prime stress-response mechanisms that are potentially conserved across cell types.

One outcome of these inducible defenses is the radiation adaptive response. A small “priming” dose (e.g., 50 mGy) triggers cellular alterations that reduce vulnerability to a subsequent higher dose. Cells exposed to such a dose have been observed, in some studies, to show reduced mutation and chromosomal aberration rates compared to unprimed cells [2,8]. This resistance may stem from upregulated DNA repair enzymes and elevated antioxidant levels. Observed across several models, including human lymphocytes and animal tissues in the 1–100 mGy range, this phenomenon suggests a dynamic protective adaptation to low-dose stress. However, it is important to note that not all studies report such an effect. Some data indicate no measurable protection or even mild adverse outcomes following low-dose priming. Moreover, where adaptive responses are observed, they are typically transient limiting their potential impact over longer time scales [3]. These findings underscore the variability of the adaptive response, which depends on dose, timing, and biological context [2].

Beyond irradiated cells, intercellular signaling amplifies effects at low doses via the radiation-induced bystander effect. Un-irradiated cells respond to signals, ROS, nitric oxide (NO), and cytokines like interleukin-8, from hit neighbors, transmitted via diffusion or gap junctions [9]. For example, studies using α-particles (a high-LET radiation type) have shown that irradiating ∼1% of cells can lead to DNA damage in neighboring cells, illustrating the bystander effect; however, such effects may differ from those observed with low-LET radiation like X-rays [10]. Antioxidant treatments (e.g., superoxide dismutase) suppress this damage, confirming ROS’s role. Bystander cells may exhibit strand breaks, micronucleus formation, or gene expression changes, though some activate protective responses like p53 and cell-cycle arrest preemptively [9]. This effect may broaden radiation’s spatial influence and raises questions about the assumptions underlying simple dose-risk scaling.

The immune system further shapes radiation outcomes, but responses are complex and vary with dose, dose rate, and exposure type. At high doses, radiation can suppress immune cell function and induce apoptosis. It may also provoke pro-inflammatory responses through cytokine release and tissue damage [11,12]. Low doses (≲100 mGy) have been reported to exhibit both immune-stimulating and anti-inflammatory effects, depending on the biological context. For example, some studies have shown enhanced natural killer cell activity or increased T-cell proliferation, while others report suppression of inflammatory signaling or mitigation of autoimmune responses [[13], [14], [15], [16], [17]]. These immune-mediated effects are often non-linear and may differ markedly between whole-body and localized exposures. Furthermore, the release of damage-associated molecular patterns (DAMPs) like ATP and fragmented DNA can activate innate immune responses through pattern recognition receptors. While these interactions may improve immune surveillance in some scenarios, potentially aiding in the clearance of pre-malignant cells, the balance between protective and deleterious outcomes remains under investigation, particularly in the very low-dose range relevant to medical imaging [[18], [19], [20]].

These processes raise important questions about the appropriateness of applying a strictly linear risk model at low doses, and have contributed to the development of the radiation hormesis hypothesis, the idea that low-level radiation (<100 mGy) may, under certain conditions, induce protective biological responses that could yield net benefits. Biphasic dose-responses in cell cultures and mammals have shown enhanced repair fidelity, reduced oxidative stress, and, in some studies, lower cancer rates compared to spontaneous levels, suggesting potential protective effects under specific conditions [21]. The hypothesis of radiation hormesis has been proposed to involve adaptive and immune mechanisms such as those discussed above, which may help offset initial damage at low doses. However, responses can vary depending on factors like genetics, dose rate, and biological context, and some studies report no evidence of a threshold for harm or even mild adverse effects instead. Although supported by specific experimental findings, hormesis remains a subject of scientific debate and ongoing investigation.

In a 2009 study, Tubiana et al. schematically summarized dose-dependent cellular responses based on an analysis of existing studies, identifying three key transition points in mammalian cells exposed to low-LET ionizing radiation: below 3 mSv, no significant signaling occurs and cells typically undergo mitotic death; between 3 and 50 mSv, error-free DNA repair predominates, with aberrant cells efficiently eliminated via apoptosis or mitotic death; between 50 and 100 mSv, cellular outcomes vary by dose rate and cell type, with increasing likelihood of DNA misrepair; and above 100 mSv, error-prone repair mechanisms become more prominent, although most damaged or preneoplastic cells are still removed [22].

## Evolution of Radiation Protection Principles and Practices

The evolution of radiation protection is rooted in our understanding of how ionizing radiation interacts with cells and the resulting biological responses. As scientific knowledge of these interactions has expanded over time, so too have the principles and practices of radiation protection, evolving from simple precautionary measures to sophisticated frameworks that balance radiation use with safety considerations. A detailed study on the history of radiation protection evolution can be found in reference [17].

The evolution of radiation protection began with scientific advances prior to the discovery of X-rays in 1895. Faraday's work on electromagnetic induction and Crookes' cathode ray tubes provided the foundation for radiation studies [23]. Simultaneously, observations of high lung cancer rates in European miners, later linked to radon exposure, marked an early recognition of radiation's potential health risks [23].

From 1895 to 1914, the discovery and widespread use of X-rays and radium introduced radiation into medical applications. Reports of injuries such as burns and dermatitis emerged shortly after the discovery with the first known instance of hand dermatitis in humans documented in January 1896 [24], though their connection to radiation was initially unclear. Early protective measures were introduced, including the use of shielding materials and limiting exposure times. By 1913, the German Roentgen Society issued the first formal recommendations, emphasizing shielding and the need for safe operational practices [25]. However, radiation measurement systems remained undeveloped, leaving gaps in effective regulation.

During World War I, advancements in X-ray technology, such as the Coolidge hot-cathode tube, allowed for more efficient and stable radiation use in medical and battlefield applications [26]. Increased awareness of occupational radiation hazards led to the introduction of time, distance, and shielding as core principles of protection. In 1915, the British Roentgen Society issued guidelines to reduce operator exposure, marking an early organized effort in radiation safety [27].

In the interwar period (1919–1938), collaboration between national and international organizations significantly advanced radiation protection. Professional societies such as the American Roentgen Ray Society and the British X-ray and Radium Protection Committee developed updated guidelines. Key milestones included the establishment of the International Commission on Radiological Units (ICRU) in 1925, which standardized radiation measurement, and the International X-ray and Radiation Protection Committee (IXRPC) in 1928, which later became the ICRP [28]. The introduction of the “roentgen” as a unit for radiation exposure and the development of dosimetry techniques, including film dosimeters and ionization chambers, enhanced monitoring capabilities [23]. Recognition of radiation’s genetic effects and health crises such as the “Radium Girls” case emphasized the need for stricter safety protocols [29].

The period from 1939 to 1945, driven by World War II and the Manhattan Project, marked the emergence of health physics as a distinct scientific discipline [27,30]. The first internal dose standards were developed, including limits on radium ingestion based on studies of radium dial painters [31]. Edith Quimby proposed a systematic approach to dose limits based on risk assessment, introducing principles such as justification, optimization, and procedural safeguards [23]. These efforts provided the foundation for post-war radiation protection practices.

From 1946 to 1960, the release of wartime research findings and the expansion of nuclear technology led to significant progress in radiation protection [32]. Concepts such as absorbed dose and dose-equivalent were introduced, enhancing the scientific basis for safety measures. The NCRP and ICRP issued detailed guidelines on whole-body exposure, critical organ protection, and internal dosimetry [30]. The ALARA (As Low As Reasonably Achievable) principle emerged, emphasizing the need to minimize radiation exposure. Advances in medical technology, such as image intensifiers and after loading techniques, reduced occupational exposure in radiology. The establishment of global organizations like United Nations Scientific Committee on the Effects of Atomic Radiation (UNSCEAR) in 1955 and the IAEA in 1957 promoted international standardization and cooperation [23].

Between the 1970s and 1990s, advancements in medical imaging technologies such as CT and PET scanning significantly impacted radiation protection [33,34]. While operator doses decreased, patient doses from these modalities increased, necessitating improved dose monitoring and optimization strategies. The ICRP and NCRP continued to revise their recommendations, incorporating new biological and epidemiological data to refine dose limits and emphasize protection principles, including ALARA and dose justification [35,36]. These developments solidified modern radiation protection practices, balancing the benefits of radiation applications with safety considerations.

The historical evolution of radiation protection demonstrates a progressive refinement of practices and principles as understanding of radiation’s biological effects expanded. From the foundational observations of radiation-related health risks in the pre-X-ray era to the rapid advancements in medical applications and nuclear technology, each milestone has contributed to the development of modern radiation safety standards. Early recognition of injuries and rudimentary protection measures laid the groundwork for organized efforts, culminating in the establishment of influential organizations like the ICRP, NCRP, UNSCEAR, and IAEA.

Throughout the 20th century, the shift from basic shielding and exposure limits to a comprehensive framework of justification, optimization, and dose limitation reflected the growing sophistication in addressing both deterministic and stochastic effects of radiation. Advances in technology, radiobiological research, and international collaboration have further strengthened safety measures, incorporating concepts such as ALARA and effective dose assessment. Modern radiation protection covers diverse applications, from medical and industrial practices to research settings, ensuring maximum benefits while minimizing risks. This continuous evolution underscores the critical role of interdisciplinary efforts and global cooperation in maintaining and advancing radiation safety in an increasingly radiation-dependent world.

## Development and Adoption of Various Risk Models Over Time

The need to approach the radiation risks systematically led to the development of various risk models. As the initial understanding was rudimentary, the first models were simple assuming that there is a direct and proportional correlation between dose and its effect. As epidemiological data from events like the atomic bombings of Hiroshima and Nagasaki became available, the approach to risk models started to refine and more sophisticated models were developed.

The LNT model for stochastic effects, which suggests that risk is directly proportional to dose without a safe threshold, gained attention and became a basis in radiation protection. Its simplicity and conservativeness made it a preferred choice for regulatory purposes, even though its validity at very low doses has been a subject of debate. As risk calculation is complicated for low doses and statistical effects do not allow real measurements for such dose values, low-dose risks need to be extrapolated.

By expanding research in this area and collecting more evidence over time, the LNT's role started to be questioned. Models suggesting sublinear responses, thresholds, and even hormetic effects, where low doses might have beneficial effects, were proposed based on various biological and epidemiological observations [37]. The Biological Effects of Ionizing Radiation (BEIR) reports, especially the BEIR VII, played a key role in shaping the scientific community's understanding and opinion on these models.

The second half of the 20th century also witnessed the introduction of the 'effective dose' concept, aiming to provide a complete measure of risk accounting for the type of radiation, the tissues exposed, and the varying sensitivities of these tissues. This concept, while invaluable in many respects, has also been the subject of question, leading to subsequent refinements and the proposal of alternative metrics like the 'effective risk.'

## Current Risk Models

**Fig. 1:**
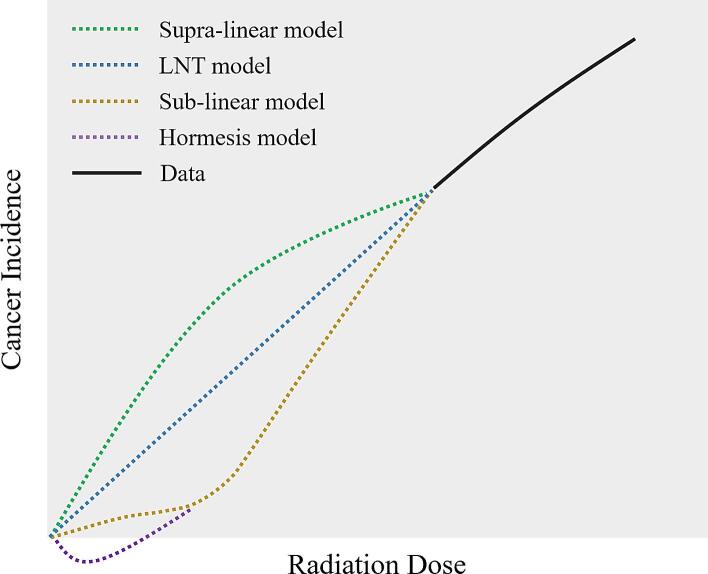
Illustration of dose–response models for radiation-induced cancer: the LNT model, which assumes risk increases linearly with dose without a safe threshold; the supra-linear model, positing higher-than-expected risk at low doses; the sub-linear (linear-quadratic) model, suggesting reduced incremental risk at lower doses due to cellular repair capacity; and the hormesis model, which hypothesizes a net protective or beneficial effect from low-dose radiation through adaptive biological responses.

The most commonly used dose-response models for estimating radiation-induced cancer risk are illustrated in Fig. 1. These models, including the LNT, sub-linear, supra-linear, and hormesis models, represent different hypotheses about how cancer incidence varies with increasing radiation dose. Each model reflects a distinct assumption about biological response mechanisms, particularly in the low-dose region, which remains the subject of ongoing scientific debate and regulatory scrutiny.

The detail of these models is as follow:

## Linear No-Threshold (LNT) Model

The Linear No-Threshold (LNT) model posits a linear relationship between radiation dose and cancer risk, with no safe threshold [38]. Favored by regulatory bodies, it is supported by robust epidemiological data and valued for its conservative, easy-to-apply nature. A summary of key studies on low-dose radiation in 2018 showed that 17% offer strong support for the LNT model, 21% moderate support, and 31% weak to moderate support; 14% are inconclusive, while 17% provide no support at all [39]. These mixed findings highlight both the substantial backing for the model and the ongoing debate surrounding its validity, particularly at very low doses. Critics argue the model may overstate risks in this range, potentially leading to unnecessary protective measures and public fear.

## Supra-linear Model

The supra-linear model suggests that cancer risk at low radiation doses may exceed the predictions of the LNT model, offering an even more conservative perspective. A supra-linear dose-response has been observed in non-radiation contexts, such as mesothelioma from asbestos exposure, where lifetime risk increases disproportionately with exposure duration [40]. However, in radiation biology, it is generally accepted that cellular repair mechanisms and immune responses help prevent significant harm at low doses [41], casting doubt on the model's broad applicability.

## Sub-linear Model (Linear-Quadratic Model)

The sub-linear model, often referred to as the linear-quadratic (LQ) model, proposes that cancer risk increases with dose but at a diminishing rate, suggesting reduced risk at low exposures compared to the LNT model. This model is grounded in radiobiological principles, particularly the LQ equation used to describe cell survival following irradiation [42]. It allows for estimating cumulative biological effects by accounting for both linear and quadratic dose components, making it useful for modeling dose-response relationships at varying dose levels.

## Hormesis Model

The hormesis model suggests that low-dose radiation may trigger beneficial biological responses, potentially reducing cancer risk compared to no exposure. This hypothesis is supported by evidence of adaptive mechanisms such as enhanced DNA repair, stress response activation, and improved immune function [43]. One key concept is the radioadaptive response, where prior low-dose exposure increases resistance to subsequent higher doses. However, these effects vary across biological systems and are influenced by genetics and environmental factors [44], limiting the generalizability of the model and highlighting the need for further research.

## Mechanistic and Individualized Models in Radiation Risk Assessment

Risk models, such as the LNT, sub-linear, supra-linear, and hormesis models, form the foundation of current radiation protection frameworks. While these models provide valuable population-level estimates of risk, they do not explicitly incorporate the biological mechanisms, such as mutation repair, clonal expansion, or immune surveillance that drive the initiation and progression of cancer. In recent years, there has been growing interest in biologically based models that attempt to describe cancer risk from ionizing radiation by explicitly incorporating mechanistic processes such as mutation induction, clonal expansion, and repopulation dynamics. One prominent example is the model developed by Schneider et al. [45,46], which accounts for the increased spread of radiation-induced mutations due to tissue repopulation after damage. This Organ Equivalent Dose (OED) framework is widely applied in radiation therapy planning to estimate second cancer risk and incorporates dose-volume effects alongside time-dependent biological processes. Unlike the LNT or LQ models, which rely on population-level dose-response extrapolations, the Schneider model bridges physical dose parameters with biological endpoints and allows for patient-specific risk projections.

Other modeling strategies, such as clonal expansion models and multistage carcinogenesis frameworks, also aim to capture the stochastic and dynamic nature of tumor development [47,48]. Though more complex and less commonly used in clinical settings, they are instrumental in research contexts for understanding how radiation alters tissue microenvironments, affects stem cell compartments, or initiates preneoplastic lesions.

These biologically informed models mark an important evolution in risk modeling by linking radiation physics with cellular and tissue-level biology. While they are not yet standard in regulatory frameworks, their continued refinement may pave the way toward more individualized, mechanism-driven radiation protection strategies.

## Uncertainties in Risk Estimations, and Challenges in Risk Communication

Radiation risk models, while widely used, have inherent limitations. No single model encompasses all the physical and biological parameters that influence radiation risk. For instance, the commonly adopted LNT model, which suggests a direct correlation between risk and dose and is supported by BEIR [49] and ICRP [50], is questioned by the French Academies report [51]. This highlights the need for a more comprehensive and universal risk model. Such a model should consider physical factors like dose rate and biological elements such as cellular repair mechanisms and genomic instability. Furthermore, to enhance accuracy, the model should be tailored to specific age and gender groups. This would address the uncertainties in conventional models that arise from generalizing across different ages and sexes. There are also challenges in accurate dose measurement, impacting the accuracy of the model that need to be considered. As an example, in internal dose measurements and estimation of the corresponding radiation risk, there are inherent uncertainties raised from phantom parameters [52]. This stem from the fact individuals is not always aligned with the model's median. Likewise, biokinetic parameters are prone to uncertainties as there are individual variances, and also data extrapolated from animal studies adds another layer of inaccuracy to the calculations when extended to human beings. The issue here is that such dosimetry uncertainties may misrepresent actual risk effects following the exposure to radiation.

Another important source of uncertainty lies in the estimation of cumulative radiation dose. These estimates, although commonly used in clinical and research contexts, are not individualized and therefore are not error-free. For example, based on a study by Durand et al. in 2012, even under standardized conditions, effective dose from CT carries an uncertainty of approximately 40% for reference phantoms [53], and when applied to actual patients, who differ in size, anatomy, and scan region, this uncertainty may increase substantially. Each step in the estimation process introduces additional variability, which accumulates and undermines the reliability of dose-based risk assessments. When such generalized dose metrics are used to estimate cancer risk, the uncertainty grows further, regardless of the accuracy with which scanner output is translated into effective dose [53]. This calls into question the clinical utility of cumulative dose and risk estimates at the individual level. Even an ideal method linking CT dose index to effective dose would leave cancer risk estimations uncertain [54]. The ICRP cautions against using effective dose for individual risk assessments from diagnostic x-rays [55]. The aggregation of multiple dose estimates in cumulative assessments can introduce more errors, increasing uncertainties.

LNT model remains a cornerstone of radiation protection, but it has recognized limitations when generalized to all exposure scenarios [56]. Dose rate is a critical modifier, and lower dose rates permit more effective DNA repair, thus reducing the biological effect per unit dose relative to high dose-rate exposures [57]. Consequently, radiological protection frameworks apply a dose and dose-rate effectiveness factor (DDREF) to scale down risk coefficients from acute high-dose data for use at low doses or low dose rates [57,58]. For example, ICRP Publication 103 retains a DDREF of 2 for solid cancers, whereas BEIR VII (2006) suggests a value around 1.5 [57,58]. Notably, leukemia risk is often modeled with a linear-quadratic dose–response, inherently reflecting a reduced slope at low doses without a separate DDREF [58].

Beyond dose-rate effects, internal exposures (e.g., inhaled radionuclides or therapeutic radiopharmaceuticals) add complexity, as radiation is delivered over extended periods and non-uniformly across tissues. Accordingly, risk from internal emitters is estimated using biokinetic and dosimetric models that track radionuclide distribution and organ doses. ICRP’s models yield dose coefficients (Sv per Bq intake) for various radionuclides, including medical isotopes, which, when combined with epidemiologically derived risk coefficients (e.g., from atomic-bomb survivor studies), enable quantification of cancer risk from internal exposures [59]. These considerations underscore the context-dependence of the LNT model’s slope, and that factors like dose rate, radiation quality (LET), and dose distribution can modulate the linear risk coefficient [58]. Indeed, UNSCEAR cautions that extrapolating high-dose findings to low-dose or internal exposures requires context-specific adjustments rather than a one-size-fits-all model [58]. Thus, accounting for dose-rate and internal-distribution effects (via DDREF and biokinetic modeling) provides a more robust, context-appropriate basis for low-dose risk estimation.

In addition, recent updates from the Life Span Study (LSS) of atomic bomb survivors suggest that the dose–response curve for solid cancers may include a significant quadratic term, especially at moderate to high doses [60,61]. This departure from strict linearity implies a curvature in risk, where the increase in cancer incidence is not proportional across the dose range. Such findings may have long-term implications for risk modeling, potentially leading to revisions of existing risk coefficients and reassessment of the LNT model’s applicability, especially in contexts involving higher cumulative exposures. While the quadratic component does not negate low-dose linear extrapolation, it reinforces the importance of refining risk models as more long-term epidemiological data become available.

Although it seems that there is a dire need for developing a more universal and comprehensive risk model, it should be noted that there are some ethical constraints on the way, apart from the technical challenges. Controlled human studies on low-dose radiation effects are ethically unfeasible due to potential harm without reasonable ground to support such a study. Consequently, researchers have to rely on observational studies, animal models, or in vitro experiments.

Ethical considerations play a significant role in communicating radiation risks. On one side, overstating these risks could discourage patients from undergoing necessary radiation-based tests [54], while understating them might lead to inadequate safety measures. It's crucial to communicate low-dose radiation risks accurately. Descriptive explanations over technical numerical values for potential cancer risks can minimize misunderstandings of the risk. We must ensure that the information about radiation risks is clear, balanced, and is supported by evidence for healthcare professionals, patients, and the broader public. This goal is not achievable unless there is an integrating knowledge from fields like epidemiology, biology, and social sciences.

## Technological and Methodological Advancements

The rapid evolution of technology and methodologies in the field of radiation risk, and consequently radiation protection, has been transformative. One of the most significant shifts has been in the domain of imaging and computational advancements. As we face the challenges associated with understanding and quantifying the risks of low-dose radiation, promising developments on the horizon offer opportunities for significant advances in this field. Modern imaging techniques have significantly advanced the way we approach radiation exposure. On one hand, with the development of advanced detector technologies and advent of new imaging modalities, we can achieve more precise imaging at lower doses, thereby reducing the radiation dose from medical procedures [62]. On the other hand, technological developments such as the one in computational power has opened new doors for better and deeper understanding of the biological processes involved in radiation exposure of living cells [63]. Complex, multi-dimensional data from biological and epidemiological studies can now be processed and analyzed with unprecedented speed and accuracy. This computational revolution can be expected to enhance our understanding of radiation biology, leading to more refined risk models, particularly in the low-dose range. However, the translation from cell-level biology to animal response to human response remains a relevant factor.

The potential of machine learning and artificial intelligence in refining risk models is particularly noteworthy [64]. These technologies can go through big datasets, identifying patterns and correlations that might be elusive to traditional analytical methods. For instance, machine learning algorithms can be trained to recognize the small signs of radiation-induced damage or to predict the likelihood of adverse outcomes based on a variety of factors. Such advancements could lead to more personalized risk assessments, tailoring radiation protection strategies to individual patient profiles with reduced uncertainty.

New research areas are further pushing the boundaries of our understanding of radiation biology and radiation risk. In the field of radiation genomics, we're trying to understand how our genes react to radiation [65]. This might help us find signs that show if someone has been exposed to radiation or if they've been harmed by it. Similarly, by studying proteins, proteomics, and our body's small molecules, metabolomics, we might see the changes radiation causes. These studies help us understand how our bodies react to small amounts of radiation, giving us a clearer insight underlying mechanism involved in predicting radiation risks.

## Insights on Radiation Risk Management

Radiation research highlights the collaborative nature of managing radiation risk, involving researchers, practitioners, and policymakers [66,67]. Studies emphasize the importance of rigorous scientific approaches in research, particularly in low-dose radiation studies where uncertainties and potential confounders are prevalent. Attention to study design and data analysis remains a critical focus. Additionally, interdisciplinary collaboration, integrating fields such as biology, epidemiology, and technology appear frequently as a means to enhance understanding of radiation risk mechanisms by addressing diverse aspects of the issue.

There is a need for practitioners to remain informed about advancements in radiation risk assessment and protection techniques. Ongoing education, potentially through workshops or training, is often noted as a way to align practices with current protocols. Across these domains, a shared emphasis on safety, particularly for patients, emerges, with radiation use in medical settings expected to serve a clear purpose.

Effective communication is another key aspect of radiation protection evident in the research. The literature points to the importance of clear, evidence-based communication that balances the benefits of radiation-based procedures with transparency about potential risks. For healthcare professionals, the ability to convey radiation risks to patients is supported by accessible informational materials and training.

These observations align with the roles of dedicated committees and organizations, as outlined earlier in this paper, which help translate such insights into actionable strategies. Their expertise ensures that research, practice, and policy evolve in a coordinated manner, guided by the latest evidence and priorities in radiation protection.

## Future Directions

As the medical community continues to recognize the cumulative risks associated with low-dose ionizing radiation, particularly in vulnerable populations such as pediatric and young adult patients, future directions in radiation protection must not only improve risk models but also embrace technological strategies that proactively reduce radiation exposure during both diagnosis and treatment.

A key area of focus is the refinement of low-dose X-ray-based imaging protocols, which remain foundational in diagnostic radiology and image-guided interventions. Techniques such as low-dose computed tomography (LDCT), particularly in lung cancer screening and pediatric imaging, are increasingly becoming standard practice. These protocols, supported by iterative reconstruction algorithms and artificial intelligence-based noise reduction, enable significant reductions in radiation exposure without compromising diagnostic accuracy.

The growing use of LDCT in population-level lung cancer screening underscores the need for careful benefit–risk evaluation. In the United Kingdom, the NHS has launched targeted lung health checks for individuals aged 55 to 74 with a history of smoking, aiming for full national rollout by 2029 [68]. In Germany, similar programs are under consideration [69]. These experiences emphasize the need for clear protocols, equitable implementation, and ongoing evaluation to ensure that the benefits of screening outweigh potential risks, including those related to cumulative radiation exposure.

These findings highlight the clinical value of LDCT in high-risk groups while underscoring the importance of optimizing protocols for patient selection, follow-up, and dose management to ensure long-term benefit and minimize cumulative radiation exposure in asymptomatic individuals.

In nuclear medicine, dose optimization remains a top priority. Advances in detector sensitivity, time-of-flight PET, and dose-reduction software now allow for lower radiotracer activity, which is especially beneficial in serial imaging and pediatric oncology settings.

Beyond diagnostics, therapeutic strategies are evolving to integrate dose-sparing technologies and improve treatment precision. A prominent example is proton therapy, which deposits the majority of its energy within the tumor via the Bragg peak, with minimal exit dose, thereby reducing the integral dose to surrounding healthy tissue. In this context, proton computed tomography (pCT) also shows promise in minimizing imaging-related exposure during treatment planning and range verification [70]. These innovations are particularly valuable in the treatment of pediatric and young adult cancers, where the long-term risks of secondary malignancies and tissue damage must be carefully managed.

Taken together, these advancements represent a shift from simply modeling and monitoring radiation risk to proactively designing clinical workflows that minimize it at the source. This forward-thinking approach, especially critical for younger populations, will be essential in aligning future clinical practice with our evolving understanding of low-dose radiation biology and long-term health outcomes.

While these clinical strategies help reduce radiation exposure in practice, progress must also continue on the theoretical front, refining how we quantify and interpret radiation risk.

Currently, risk estimation largely relies on the LNT model, which assumes a direct, proportional relationship between dose and risk. Although widely accepted and supported by several regulatory bodies, the LNT model remains contested, particularly at very low doses where epidemiological data are limited. Despite its limitations, the LNT model is considered a practical and cautious framework for radiation protection [39]. As more real-world and low-dose data become available, it is expected that this model, alongside emerging alternatives, will evolve to better reflect actual risk.

At the biological level, important gaps remain in our understanding of the cellular response to low-dose radiation [71]. Future research should aim to clarify the persistence of DNA damage and the role of the immune system in modulating cancer risk following exposure. A more detailed understanding of these mechanisms will provide a stronger foundation for risk modeling and mitigation strategies.

Looking ahead, the availability of large, well-characterized epidemiological datasets is expected to significantly improve our understanding of long-term radiation risks, particularly the risk of second cancers. As follow-up data collection becomes more systematic in several countries, clinical studies will increasingly provide more reliable outcome measures related to low-dose exposures. Notable efforts such as the Million Worker Study offer valuable benchmarks for assessing dose–response relationships across diverse populations [72]. These studies, by incorporating occupational and medical exposure data alongside health outcomes, will allow for more accurate estimation of risk coefficients and may contribute to refining existing models like LNT. Furthermore, they can support the stratification of risk by variables such as age, sex, dose rate, and exposure type, addressing many of the uncertainties that currently limit individualized risk assessment.

Finally, the integration of machine learning and artificial intelligence offers promising opportunities for improving radiation risk assessment. These technologies can identify subtle patterns in large datasets, discover biomarkers of radiation exposure, and support more individualized risk predictions. Such innovations could fundamentally transform how we evaluate radiation risk, shifting from generalized estimates to personalized, data-driven insights.

Ultimately, addressing radiation risk requires a multidisciplinary approach. Collaboration between epidemiologists, biostatisticians, data scientists, clinical practitioners, and policymakers will be key to developing comprehensive models that account for both the biological effects of radiation and its societal implications. Only through such collaborative efforts, can we reduce uncertainty and advance toward safer, more effective use of ionizing radiation in medicine.

## Conclusion

From its early days, radiation protection and radiation risk has witnessed significant evolutions. Several risk models like the LNT as well as supra-linear, sub-linear, and hormesis models have been developed that helped us take serious steps to quantify the risks of radiation exposure. These models helped us to understand the potential risks involved in radiation-based procedures. The LNT model, considered the strongest, is practical and supported by several studies. However, some studies are not in line with this model, indicating the need for continued research in this area. The journey continues and there are research gaps that need to be filled to help us reach a model with reduced uncertainties compared to the current models. In this context, the potential of interdisciplinary collaborations and tapping into technological advancements can help us to take promising steps to reach such a refined model [73]. As we are receptive to new findings, we should also pay a special attention to transparent communication and how the risk of the radiation is communicated amongst the professionals and to the general public.

## CRediT authorship contribution statement

**Forough Jafarian-Dehkordi:** Visualization, Investigation, Data curation, Methodology, Formal analysis, Conceptualization, Writing. **Christoph Hoeschen:** Review and Editing.

## Declaration of competing interest

The authors declare that they have no known competing financial interests or personal relationships that could have appeared to influence the work reported in this paper.
